# Zearalenone Changes the Diversity and Composition of Caecum Microbiota in Weaned Rabbit

**DOI:** 10.1155/2018/3623274

**Published:** 2018-10-08

**Authors:** Peng Li, Shuhua Yang, Xin Zhang, Sheng Huang, Nan Wang, Mingyang Wang, Miao Long, Jianbin He

**Affiliations:** Key Laboratory of Zoonosis of Liaoning Province, College of Animal Science & Veterinary Medicine, Shenyang Agricultural University, Shenyang 110866, China

## Abstract

Mycotoxins exhibit several severe effects on intestinal health, but few studies have assessed mycotoxins effect on the intestinal microflora and its repercussions to humans and animals. In this study, we evaluated the effect of zearalenone (ZEA), one of the most harmful mycotoxins on the structure of caecal microbiota in rabbits. Twenty-eight male weaned rabbits were randomly divided into four groups and orally given different concentrations of ZEA (400, 800, and 1600 *μ*g/kg.b.w). Microbial communities in caecum samples of rabbits were analyzed for 16S rRNA by Illumina sequencing through Illumina Miseq platform after being fed for 28 days. The results showed that increasing ZEA doses increased the species richness but did not significantly increased the species diversity of the caecum microbiota in the rabbits. In addition, the caecum microbiota from the samples in different ZEA-treated groups was clustered according to their dosing regimens. At the phylum level, ZEA decreased the abundance of* Actinobacteria* and significantly increased the abundance of* Cyanobacteria*,* Synergistetes*, and* Proteobacteria*. At the genus level, there were declines in the abundance of* Adlercreutzia*,* Blautia*,* Desulfitobacter*,* Lactobacillus*,* Oxalobacter*, and* p-75-a5*. The decrease of abundance in* Lactobacillus*,* Desulfitobacter*, and* p-75-a5* was particularly noticeable. In conclusion, zearalenone could increase *α*-diversity but significantly decrease the abundance of some bacteria with the important metabolic functions. These findings suggested that ZEA could modify the caecum microbiota.

## 1. Introduction

There is a complex population of microbes resides in the gastrointestinal tract, and these microbes are critical to the healthy development of the immune system and animal health [1] (Schuijt et al., 2013). Some microbiota in gastrointestinal tract can produce antimicrobials and form a barrier against pathogens and play multiple important roles in keeping the intestinal morphology, improving digestion, and modulating the host gene expression [2, 3]. Recently, studies have shown that intestine microbiota is involved in multiple health problems such as obesity and inflammatory bowel disease [4, 5]. Therefore, maintaining the balance of intestinal flora or regulating the intestinal flora such as increasing the abundance of beneficial microorganisms is very meaningful for animals and human health. However, the intestinal flora can be affected by many factors, such as diet, environment, age, pathogenic bacteria, and some xenobiotic [6–9].

Mycotoxins are secondary metabolites produced by fungal genera that are toxic, carcinogenic, and/or teratogenic, resulting in significant adverse effects food safety and public health [10, 11]. Among the mycotoxins, zearalenone (ZEA, also known as F-2 toxin), a nonsteroidal estrogenic mycotoxin produced by various Fusarium species [12], is considered as a common contaminant of food and feedstuffs [13]. Many studies have demonstrated that ZEA can affect the immunologic function, influence the liver and kidney function, and severely impact on reproductive system in mice [14]. Many studies have confirmed that the main mechanism of ZEA injuring the body is due to its ability to induce high estrogen effects, endoplasmic reticulum stress, and activation of mitochondrial apoptosis [15–17].

There have been many reports about the toxic mechanism of mycotoxins. However, few studies have reported for mycotoxins with demonstration of effects on the intestine microbiota [18, 19]. The effects of only a few types of mycotoxins on intestine microbiota have been studied so far: aflatoxin B1 [20–22], zearalenone [23] deoxynivalenol [24–27], fumonisin B1 [28, 29], and ochratoxin A (Guo et al., 2010). These researches give us some important knowledge that microbiota are key targets for dietary mycotoxins and contributes to host resistance to them and mycotoxins can modify the composition of microbial communities.

There are few reports about the effect of ZEA on intestinal microflora, especially on the intestinal flora of rabbit. Therefore, the effect of ZEA on the cecum microflora in rabbits is studied in this study. This study will expand a knowledge of the effects of mycotoxins on the intestinal microflora.

## 2. Materials and Methods

### 2.1. Animals and Treatment

The 28 weaned New Zealand rabbits (aged 50 days, body mass 2.2kg ± 0.2kg) were bred in a room at a temperature ranging from 22 to 24°C and the rabbits were subjected to an atmosphere with a relative humidity of between 40 to 60%. Water and diet were provided* ad libitum *for the rabbits. The rabbits were acclimatised for one week after transportation. The experimental procedures have been approved by the Ethics Committee for Laboratory Animal Care (Animal Ethics Procedures and Guidelines of the People's Republic of China) for the use of Shenyang Agricultural University, China (Permit No. 264 SYXK<Liao>2011-0001).

Rabbits were randomly distributed into four groups, and each group had seven rabbits and the rabbits in each group were in one cage. Animals within different treatment groups were treated daily by oral gavage at 14:00 for 28 days. The four groups are as follows: control group, administrated with control vehicle (DMSO); the low dose group (400*μ*g/kg, b.w., ZEA), the middle dose group (800*μ*g/kg, b.w., ZEA); the high dose group (1600*μ*g/kg, b.w., ZEA). The concentration of zearalenone was adapted daily according to the changes of rabbit weight. DMSO was used due to the hydrophobicity of ZEA and its minimum effects on animals (<50*μ*L/animal) [20]. The rabbits were sacrificed, 24 h after final treatment, under anaesthesia. The cecal contents were taken sterilely and stored in a freezer bag at -80 C refrigerator.

### 2.2. DNA Extraction

Total bacterial genomic DNA samples were extracted using the Fast DNA SPIN extraction kits (MPBiomedicals, Santa Ana, CA, USA), following the manufacturer's instructions, and stored at -20°C prior to further analysis. The quantity and quality of extracted DNAs were measured using a NanoDrop ND-1000 spectrophotometer (Thermo Fisher Scientific, Waltham, MA, USA) and agarose gel electrophoresis, respectively.

### 2.3. 16S rDNA Amplicon Pyrosequencing

PCR amplification of the bacterial 16S rRNA genes V3–V4 region was performed using the forward primer 338F (5'-ACTCCTACGGGAGGCAGCA-3') and the reverse primer 806R

(5'-GGACTACHVGGGTWTCTAAT-3'). Sample-specific 7-bp barcodes were incorporated into the primers for multiplex sequencing. The PCR components contained 5 *μ*l of Q5 reaction buffer (5×), 5 *μ*l of Q5, High-Fidelity GC buffer (5×), 0.25 *μ*l of Q5 High-Fidelity DNA Polymerase (5U/*μ*l), 2 *μ*l (2.5 mM) of dNTPs, 1 *μ*l (10 uM) of each Forward and Reverse primer, 2 *μ*l of DNA Template, and 8.75 *μ*l of ddH2O. Thermal cycling consisted of initial denaturation at 98°C for 2 min, followed by 25 cycles consisting of denaturation at 98°C for 15 s, annealing at 55°C for 30 s, and extension at 72°C for 30 s, with a final extension of 5 min at 72°C. PCR amplicons were purified with Agencourt AMPure Beads (Beckman Coulter, Indianapolis, IN) and quantified using the PicoGreen dsDNA Assay Kit (Invitrogen, Carlsbad, CA, USA). After the individual quantification step, amplicons were pooled in equal amounts, and pair-end 2×300 bp sequencing was performed using the Illlumina MiSeq platform with MiSeq Reagent Kit v3 at Shanghai Personal Biotechnology Co., Ltd (Shanghai, China).

### 2.4. Sequence Analysis

The Quantitative Insights into Microbial Ecology (QIIME, v1.8.0) pipeline was employed to process the sequencing data. Briefly, raw sequencing reads with exact matches to the barcodes were assigned to respective samples and identified as valid sequences. The low-quality sequences were filtered through following criteria: sequences that had a length of <150 bp, sequences that had average Phred scores of <20, sequences that contained ambiguous bases, and sequences that contained mononucleotide repeats of >8 bp. Paired-end reads were assembled using FLASH. After chimera detection, the remaining high-quality sequences were clustered into operational taxonomic units (OTUs) at 97% sequence identity by UCLUST (Edgar 2010). A representative sequence was selected from each OTU using default parameters. OTU taxonomic classification was conducted by BLAST searching the representative sequences set against the Greengenes Database using the best hit. An OTU table was further generated to record the abundance of each OTU in each sample and the taxonomy of these OTUs. OTUs containing less than 0.001% of total sequences across all samples were discarded. To minimize the difference of sequencing depth across samples, an averaged, rounded rarefied OTU table was generated by averaging 100 evenly resampled OTU subsets under the 90% of the minimum sequencing depth for further analysis.

### 2.5. Bioinformatics and Statistical Analysis

Sequence data analyses were mainly performed using QIIME and R packages (v3.2.0). OTU-level alpha diversity indices, such as Chao1 richness estimator, ACE metric (Abundance-based Coverage Estimator), Shannon diversity index, and Simpson index, were calculated using the OTU table in QIIME. OTU-level ranked abundance curves were generated to compare the richness and evenness of OTUs among samples. Beta diversity analysis was performed to investigate the structural variation of microbial communities across samples using UniFrac distance metrics and visualized via principal coordinate analysis (PCoA), nonmetric multidimensional scaling (NMDS), and unweighted pair-group method with arithmetic means (UPGMA) hierarchical clustering. Differences in the Unifrac distances for pairwise comparisons among groups were determined using Student's t-test and the Monte Carlo permutation test with 1000 permutations, and visualized through the box-and-whiskers plots. The taxonomy compositions and abundance were visualized using MEGAN and GraPhlAn. Venn diagram was generated to visualize the shared and unique OTUs among samples or groups using R package “VennDiagram,” based on the occurrence of OTUs across samples/groups regardless of their relative abundance. Taxa abundance at the phylum, class, order, family, genus, and species levels was statistically compared among samples or groups by Metastats and visualized as violin plots.

### 2.6. Statistical Analysis

The one-way ANOVA method was used to analyze the data with SPSS 19.0 program, and Tukey's post hoc test was evaluated for significance difference (p < 0.05; p < 0.05). Data were presented as the mean ± standard deviation.

## 3. Results

### 3.1. Animal Pathology

The weights of rabbits in the high-dose and middle-dose groups were significantly lower than that in control group at the end of the four-week experiment (p < 0.01). We assessed the kidneys to confirm the reliability of our ZEA-induced rabbits model. The weights of liver and kidney and their indexes significantly decreased in the ZEA group compared with the control group (p < 0.05). Histopathological examination demonstrated that ZEA at high–doses caused lobulation and atrophy of the glomerulus in murine kidneys and multiple inflammatory cells with focal infiltration in the liver. These characteristics are consistent with the effects of high-dose zearalenone on animal performance and pathological damage.

### 3.2. DNA Sequence Data

**Figure 1:**
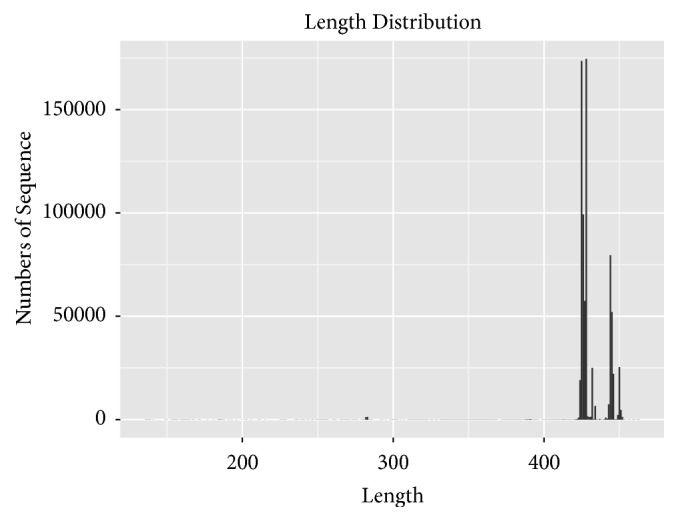
Fragment length distribution of sequences from each sample after merging and trimming.

**Figure 2:**
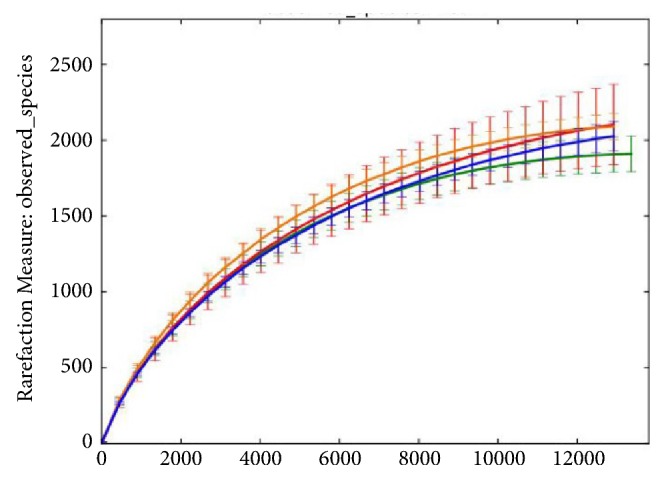
Rarefaction curves of the OTUs number at 97% similarity box plot for every sample. Green, blue, orange, and red indicate the 7 samples of control, low dose ZEA-treated group, middle dose ZEA-treated group, and high dose ZEA-treated group, respectively.

From the cecal contents of the 28 rabbits sequencing analysis through a high-throughput sequencing on the Illumina MiSeq platform, the original sequence was obtained after the quality control of a total of 1432539 valid sequences and an average of 51162 sequences per sample. Among the high-quality sequences, about 99.94% were longer than 400 bp and most were between 420 and 460 bp (Figure 1). Rarefaction analysis results showed that this sequencing depth was sufficient to cover the microbial diversity of each sample (Figure 2).

### 3.3. Microbiological Taxonomy Analysis

According to the OTU classification and the results classification status identification, the specific composition and bacterial flora abundance map of each sample at the level of the phylum class, order, family, genus, and species were obtained by using QIME software.

**Figure 3:**
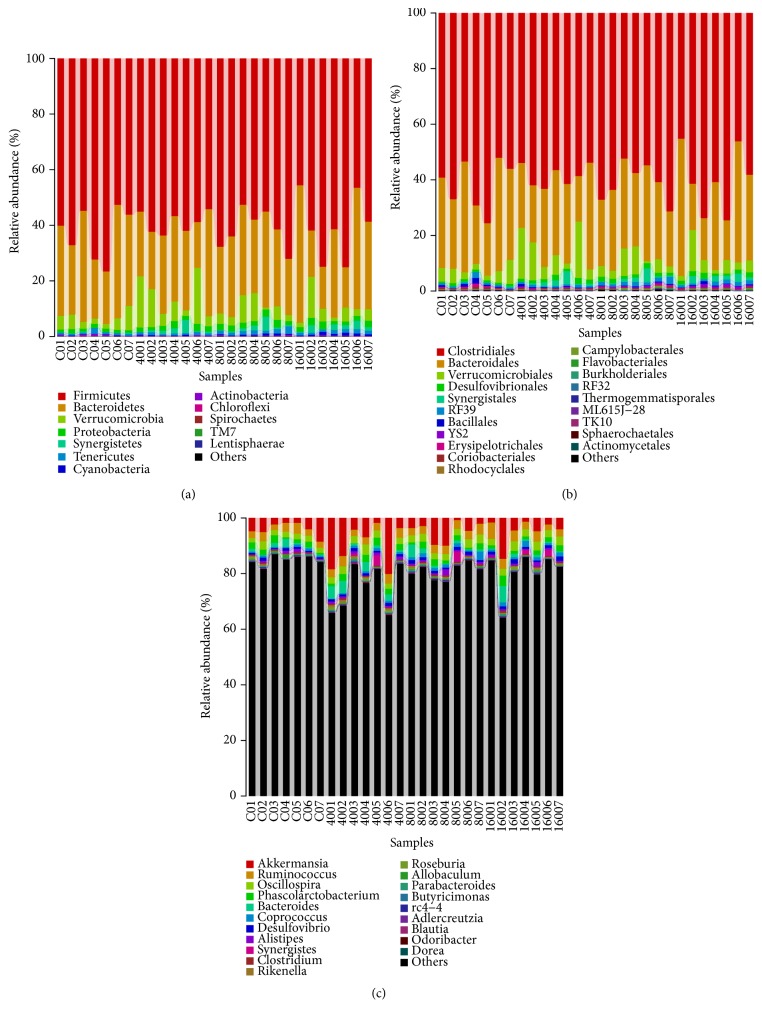
Relative abundance of the main bacterial communities found in each samples at Flylum level (a), Order level (b), and Genus level (c). C01-C07 represents the control group; 4001-4007 represent the low dose ZEA-treated group; 8001-8007 represent the middle dose ZEA-treated group; 16001-16007 represent the high dose ZEA-treated group.

The taxon abundance of each sample was identified into 12 phyla, 19 classes, 23 orders, 39 families, 60 genera, and 68 species in our study groups. In this study, the distributions of bacterial composition at the phylum, order, and genus levels are shown in Figures 3(a), 3(b), and 3(c). Among them, four phyla (*Firmicutes, Bacteroidetes, Verrucomicrobia, *and* Proteobacteria*) were commonly found in each group, which account for about 61.0%, 28.3%, 5.9%, and 1.8%, respectively (Figure 3(a)). At the order level,* Clostridiales, Bacteridales, verrucomicrobiales, *and* Desulfovibrionales *were commonly found in each group, which account for 60.3%, 28.3%, 5.9%, and 1.3%, respectively (Figure 3(b)). At the genus level,* Akkermansia*,* Ruminococcus, Oscillospiva, phascolarctobicterium, Bacteroidales, Coprococcus, *and* Desulfovibrio* were commonly found in each group (Figure 3(c)).

### 3.4. Alpha Diversity of the Ceacum Microbiota

**Figure 4:**
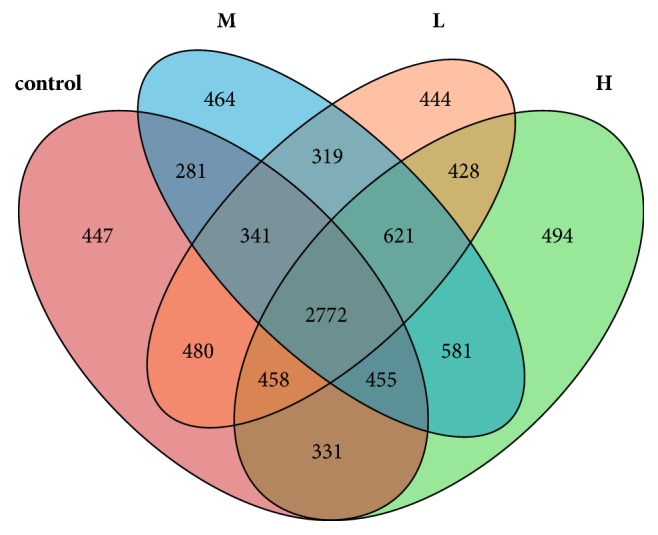
Venn diagram summarizing the numbers of common and unique OTUs (3% distance level) among the four groups. Each* circle *represents a set of samples, the group between the circle and circle overlapping part digital represent of the common OTUs, and there is no overlapping part representing unique OTUs in each group. Pink represents the control group; blue represents the low dose ZEA-treated group; orange represents the middle dose ZEA-treated group; green represents the high dose ZEA-treated group.

The ACE, Chao1, Shannon, and Simpson indexes can indicate microbial diversity and species richness [30, 31]. As shown in Table 1, the Shannon and Simpson indexes indicate that the species diversity was an upward trend with the increase of the concentration of ZEA, but the difference was not significant (p > 0.05) and the results indicated that ZEA could not significantly change the species diversity; gowever, the ACE and Chao 1indexes were difference between group of control and group of 400*μ*g/kg ZEA (p < 0.05); significant difference between group of control and group of 800*μ*g/kg ZEA (p < 0.01); and difference between group of 800*μ*g/kg ZEA and group of 1600*μ*g/kg ZEA (p < 0.05); the ACE and Chao1 indexes indicated that species richness in all ZEA-treated groups were higher than that in the control group, and the results indicated that ZEA could significantly change the species richness. Venn diagrams were used to evaluate the distribution of OTUs among the different treatment groups. As shown in Figure 4, the Venn diagram displayed that the 5565 OTUs, 5834 OTUs, 5863 OTUs, and 6140 OTUs were identified from the samples in control, low, middle, and high ZEA-treated group, respectively; the number of OTUs was higher in low, middle, and high dose ZEA-treated group than in the control group; and the total of 2772 OTUs was identified as constituting core bacterial OTUs in the four groups (Figure 6). The number of unique OTUs was 447 (control), 444 (L), 464 (M), and 494 (H), in each group, respectively. There were 621 OTUs shared in ZEA groups.

### 3.5. Beta Diversity of the Ceacum Microbiota

**Figure 5:**
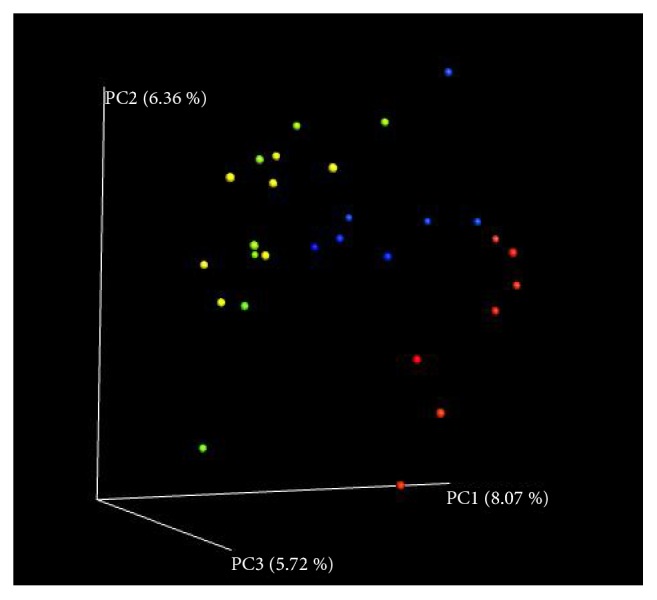
The principal co-ordinates analysis (PCoA) with Unweighted Unifrac Distances of the rabbits caecum microbiota. The percentage represents contribution of principal component to the difference of samples. Each symbol represents each gut microbiota. red dot, the control group; blue dot, the low dose ZEA-treated group; yellow dot, the middle dose ZEA-treated group; green dot, the high dose ZEA-treated group. The points of different colors belong to different samples (groups). Each point represents one sample. The closer of the distance between two points means that the higher of the similarity and the smaller the difference of the microbial community structure between the two samples.

**Figure 6:**
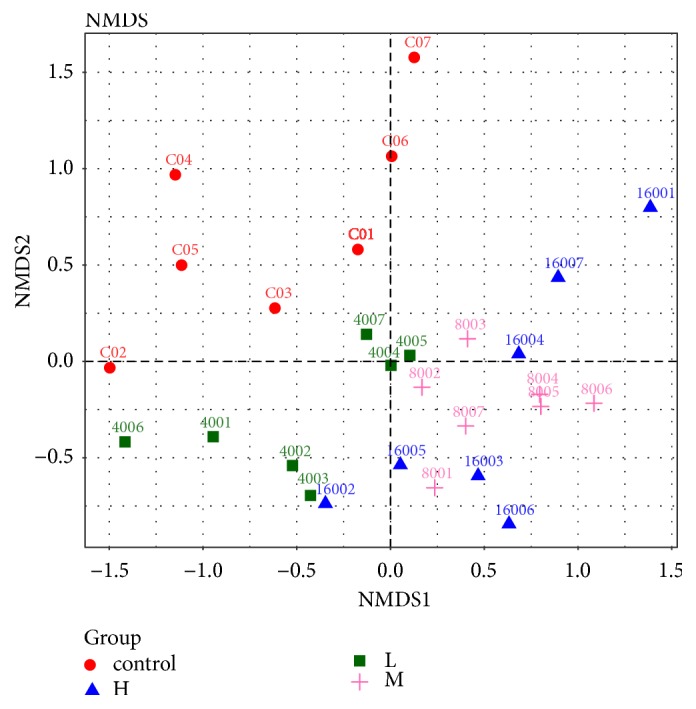
Multiple samples NMDS analysis of the rabbits caecum microbiota. Red circle, the control group; green square, the low dose ZEA-treated group; pink cross; the middle dose ZEA-treated; blue triangle, the high dose ZEA-treated group. The points of different colors belong to different samples (groups). Each point represents one sample. The closer of the distance between two points means that the higher of the similarity and the smaller the difference of the microbial community structure between the two samples.

**Figure 7:**
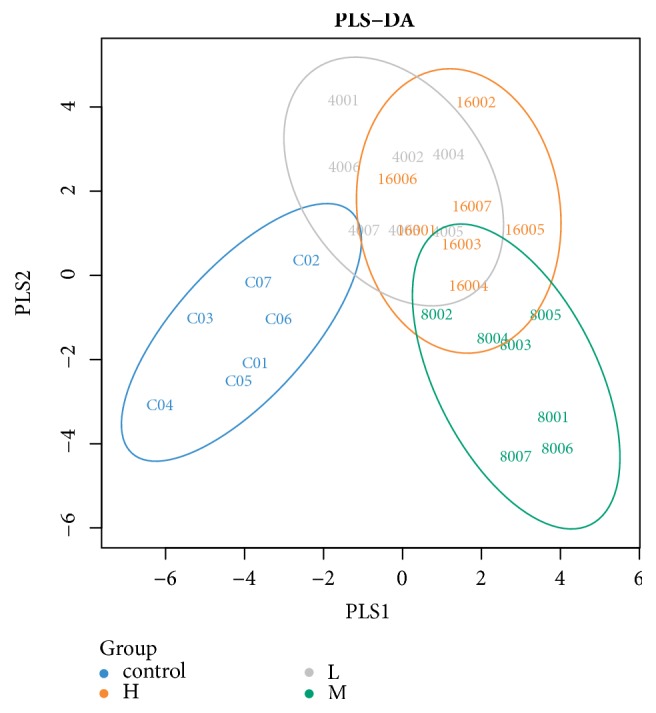
The effect of zearalenone on the microbial community structure of caecum in rabbits by using PLS-DA (Partial Least Squares Discriminant Analysis) methods. Each point represents a sample, points of the same color belong to the same group, and points of the same group are marked with ellipses. If the samples belonging to the same grouping are closer to each other and the distance between the points of different grouping is farther, the classification model is better.

A beta diversity map based on PCoA Analysis with Unweighted Unifrac Distances (Figure 5), NMDS nonmetric multidimensional scale analysis with UniFrac distance (Figure 6), and Partial Least Squares Discriminant Analysis (Figure 7) showed that the similarity in species diversity is very different when ZEA was given to the rabbits.

As shown in Figures 5, 6 and 7, there was a clear distance of rabbit's caecum flora between the groups of the high-dose, middle-dose ZEA treatment with the control group, which indicated that the rabbit's caecum flora structure was changed when the rabbits were treated with the high-dose, middle-dose ZEA. It also showed that there was no difference of the distance of rabbit's caecum flora between the high-dose treatment group with the middle-dose treatment group, which indicated that there was little difference of rabbit's caecum flora between the high-dose group and the middle-dose group. We also found that the group spacing in the ZEA treatment groups was smaller than that between the ZEA treatment group with the control group, which indicated that the difference of the rabbit's caecum flora structure between the ZEA treatment groups was smaller than that between the ZEA treatment groups with the control group. ZEA at high dose could significantly change the species diversity of bacteria in caecum of rabbits, although data showed that the contribution of the three mains components is weak (all>10%).

### 3.6. Analysis of Diversity of Samples

**Figure 8:**
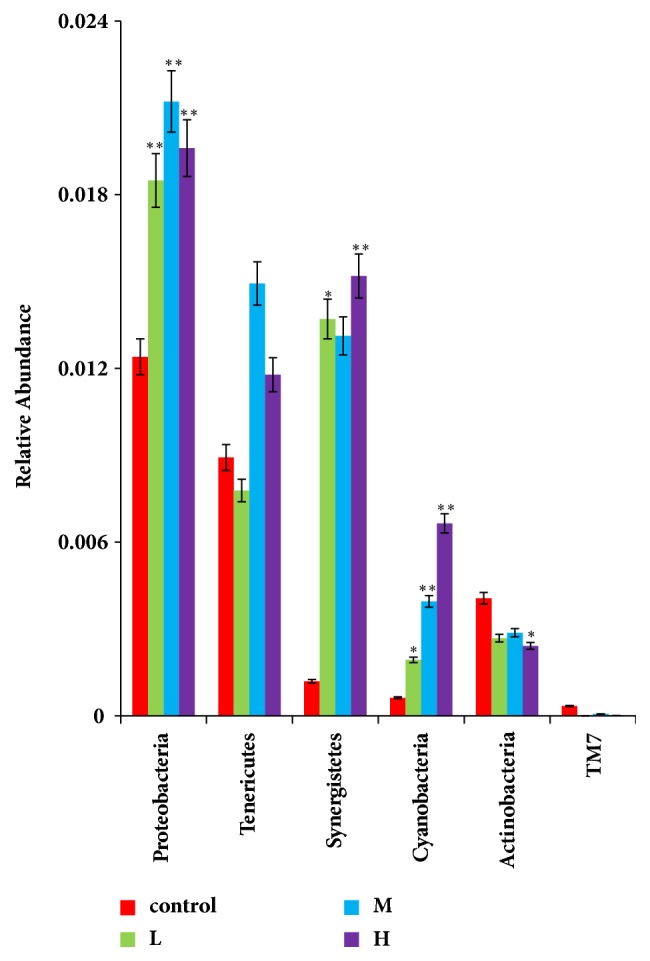
The significant different microbiota abundance in phylum level with the increase of the concentration of ZEA. The abscissa of the figure was groups and ordinate was taxa abundance. Red, the control group; green square, the low dose ZEA-treated group; blue; the middle dose ZEA-treated; purple, the high dose ZEA-treated group; *∗*p < 0.05 vs. control group; *∗∗*p < 0.01 vs. control group.

As it shown in Figure 8, at the phylum level, compared with the control group, the abundance of Cyanobacteria was significantly increased in all ZEA-treated group (p<0.05); the abundance of* Synergistetes* was increased in low level and high level ZEA-treated group (p<0.05; p<0.01); the abundance of* Proteobacteria* was also increased in low level, middle level, and high level ZEA-treated group (p<0.05; p<0.01). However, the abundance of* Actinobacteria* was decreased in high level ZEA-treated group (p<0.05). The results indicated that, with the increase of ZEA concentration, ZEA decreased the abundances of* Actinobacteria* and significantly increased the abundances of* Cyanobacteria, Synergistetes, *and* Proteobacteria *(p<0.05; p<0.01).

**Figure 9:**
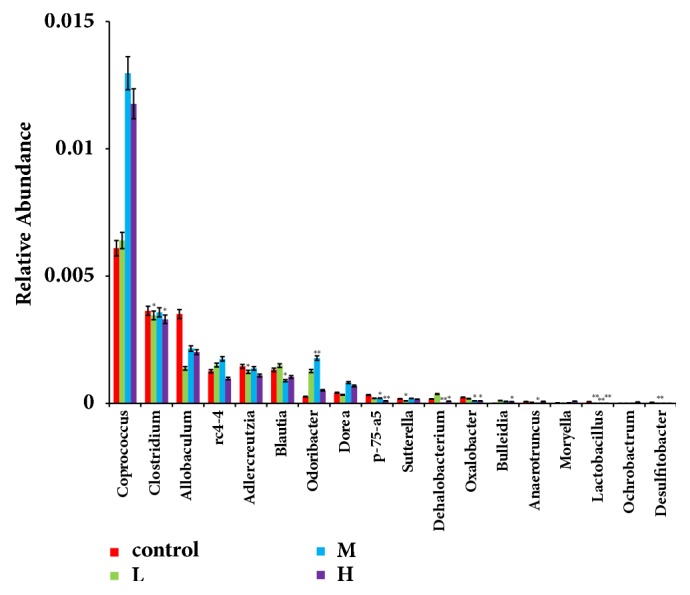
The significant different microbiota abundance in genus level with the increase of the concentration of ZEA. The abscissa of the figure was groups and ordinate was taxa abundance. Red, the control group; green square, the low dose ZEA-treated group; blue; the middle dose ZEA-treated; purple, the high dose ZEA-treated group; *∗*p < 0.05 vs. control group; *∗∗*p < 0.01 vs. control group.

As shown in Figure 9, at the genus level, compared with the control group, there are eight significant different kinds of abundance of the caecum microflora in ZEA-treated group:* Adlercreutzia, Blautia, Dehalobacterium, Desulfitobacter, Lactobacillus, Oxalobacter, p-75-a5,* and* Ochrobactrum*. With the increase of ZEA concentration, ZEA decreased the abundance of* Adlercreutzia, Blautia, Desulfitobacter, Lactobacillus, Oxalobacter,* and* p-75-a5* (p<0.05; p<0.01) and particularly significantly decreased the abundances of* Lactobacillus* (p<0.01).

## 4. Discussion

Recent researches have displayed that some mycotoxins such as AFB1, OTA, DON can modulate the intestinal bacterial community compostionin in pig or rat [20, 24, 27, 32]. However, there are no reports about ZEA effect on the caecum microflora in weaned rabbits. So our study can enrich the knowledge of the effect of mycotoxins on the intestinal flora.

In this study, we selected the weaned rabbits, because the composition of rabbit intestinal flora tended to be stabilized [33, 34], and the caecal microbiota developed progressively from a simple and unstable community after birth into a complex and climax community in subadult rabbits [35]. So we chose the rabbits after weaning to carry out the animal experiment and could more accurately and truly react to effect of ZEA on the cecum flora of the rabbits.

Our results showed that the main phyla were Firmicutes, followed by Bacteroidetes in caecum bacterial communities of rabbits, in which results were in accordance with previous studies on the caecum microbiota of rabbits [31, 36, 37]. Our results found that compared with the control group, ZEA did not affect the abundance of* Bacteroidetes* and* Firmicutes*. However, our results showed that ZEA significantly increased caecum* Proteobacteria* phylum (Figure 9). Although* Proteobacteria*, a minor constituent within the hindgut microbial community in rabbits [38], accounted for only 1.8% in ceacum of rabbits (Figure 3), it included many pathogenic bacteria, such as* Escherichia coli*,* Salmonella *and* Vibrio cholera*; thus, an increased abundance of* Proteobacteria *was relate to severe intestinal inflammation, such as bowel disease and necrotizing enterocolitis [39], which was potential diagnostic microbial signature of epithelial dysfunction [40]. We hypothesize that ZEA might increase some bacteria in flylum* Proteobacteria* then cause the intestinal inflammation. However, further research is needed to prove it.

Our results found that the abundance of phyla* Cyanobacteria* in caecum was significantly increased after the rabbits administrated with ZEA. Previous studies showed that phylum* Proteobacteria *was found in the small or large intestine of sheep, cattle, and pigs [41–43] and in infant feces [44]. Little is known about the functions of Cyanobacteria and their effect on the bacterial communities in the mammalian gut. Some research showed that some species in* Cyanobacteria *has some functions such as obligate anaerobic fermentation, syntrophic H_2_-production, production of oxygen, nitrogen fixation, and synthesis of vitamin B and K21 in nature [45]. However, some research found severe hyperplasia of intestinal epithelium of fish after cyanobacterial exposure because some species in* Cyanobacteria* can produce toxic metabolites known as cyanotoxins [46]. Therefore, it is difficult to speculate the consequences of the increase of cyanobacterial abundance caused by ZEA, if we do not know exactly which bacterial abundance changes, and further studies are needed.

Our results also showed that ZEA significantly increased the abundance of phyla* Synergistetes*.* Synergistetes*, one of the opportunistic bacteria, species within this phylum have also been implicated in periodontal disease, gastrointestinal infections, and soft tissue infections [47–50]. Therefore, we predict that the increase of the abundance of* Synergistetes* caused ZEA to have dealeterious effects on the intestinal health of the rabbits.

The abundance of genus* Lactobacillus* was significantly decreased in all ZEA-treated groups. These results were consistent with some reports that* Lactobacillus* significantly depleted by ZEA, AFB1, and DON [27, 51, 52]. The species in genus* Lactobacillus* are considered to be the most important probiotic in the intestinal tract, which can adhere to intestinal epithelial cells and then reduce the destruction of epithelial cells by pathogenic bacteria. These species also can bind and remove ZEA via some composition of their cell surface [53–55]. We speculated that ZEA reduced the abundance of genus* Lactobacillus *which might be because when it was adsorbed on the surfaces of* Lactobacillus*, ZEA could damage their cell wall and then cause* Lactobacillus* death then removed from the intestine. Another reason we guess might be due to that antimicrobial activities of ZEA to gram-negative and -positive bacteria. However, there is no relevant reports about ZEA have the antimicrobial activities.

Studies showed that genera and* Clostridium* and* Blautia* are common constituents of healthy adult gut microbiota [56]. Most of species in genus* Clostridium are responsible for producing butyric acid* [57], and Blautia sp. are have the ability to metabolize flavonoids and utilize carbohydrates as fermentable substrates, which play important roles in the digestion of the diets in cecum [58, 59]. The genus Adlercreutzia only has one species, namely,* A. equolifaciens*, which can produce equol [60]. The genus Oxalobacter commonly inhabits the intestine and can degrade oxalate as its major energy [61] (Liu et al., 2016). The desulfitobacter spp. can dehalogenate halogenated organic compounds by mechanisms of reductive dehalogenation [62]. Therefore, these above genus bacteria play an important metabolic function in the intestine and can significantly affect the intestinal digestion. Combined with the results ZEA significantly reduces the abundance of these genera and we predicted that ZEA severely affects the intestinal flora balance in rabbits and then affects the intestinal digestion.

## 5. Conclusions

Until now, the mechanism by which ZEA affects intestinal microflora is still unclear. However, for the first time, we studied the effect of ZEA on the caecum microflora of weaned rabbits and concluded that ZEA could significantly affect the balance of caecum microflora and reduce the abundance of some bacteria with important metabolic function. We speculate that the effects of ZEA on intestinal microflora will affect the intestine digestion function and health of the rabbits, but it needs to be further confirmed.

## Figures and Tables

**Table 1:** Microbial diversity indices in different treatment groups.

**groups**	**Shannon**	**Simpson**	**Chao1**	**ACE**
**control**	0.9799±0.0086	8.79±0.34	1911.82±78.77	1938.26±96.64
**400** ***μ*** **g/kg ZEA**	0.9844±0.0124	9.17±0.40	2108.41±74.13^*∗*^	2120.73±88.66^*∗*^
**800** ***μ*** **g/kg ZEA**	0.9825±0.0056	8.83±0.17	2358.64±258.19^*∗∗*^	2440.92±280.98^*∗∗*^
**1600** ***μ*** **g/kg ZEA**	0.9850±0.0112	8.80±0.64	2537.89±393.02^*∗∗*#^	2605.26±399.38^*∗∗*#^

One-way ANOVA and Tukey's post hoc test were employed to assess the significance of differences between the four groups. The ACE and Chao 1 indexes represent the community richness of the microbiota, and the Shannon and Simpson indexes represent the community diversity of the microbiota. ^*∗*^p < 0.05 vs. control group; ^*∗∗*^p < 0.01 vs. control group; ^#^p < 0.05 vs. 400*μ*g/kg ZEA.

## Data Availability

The data used to support the findings of this study are available from the corresponding author upon request.
